# Structured pain-free exercise progressively improves ankle-brachial index and walking ability in patients with claudication and compressible arteries: an observational study

**DOI:** 10.1007/s11739-021-02827-4

**Published:** 2021-09-09

**Authors:** Fabio Manfredini, Luca Traina, Vincenzo Gasbarro, Sofia Straudi, Lorenzo Caruso, Fabio Fabbian, Paolo Zamboni, Roberto Manfredini, Nicola Lamberti

**Affiliations:** 1 Department of Neuroscience and Rehabilitation, University of Ferrara, Via Luigi Borsari 46, 44121 Ferrara, Italy; 2 Unit of Physical and Rehabilitation Medicine, University Hospital of Ferrara, Ferrara, Italy; 3 Unit of Vascular and Endovascular Surgery, University Hospital of Ferrara, Ferrara, Italy; 4 Department of Medical Sciences, University of Ferrara, Ferrara, Italy; 5 Vascular Diseases Center, University Hospital of Ferrara, Ferrara, Italy

**Keywords:** Peripheral artery disease, Cardiovascular risk, Atherosclerosis, Physical exercise, Blood pressure, Ankle-brachial index

## Abstract

In patients with peripheral artery disease (PAD), supervised exercise at near-moderate pain improves walking ability but not ankle-brachial index (ABI) values. In a retrospective observational study, we determined vascular and functional effects of a 6-month structured pain-free exercise program in patients with claudication and compressible vessels. Four-hundred and fifty-nine consecutive patients were studied. Segmental limb pressures were measured and ABI calculated during circa-monthly hospital visits. The 6-min (6MWD) and the pain-free walking distance (PFWD) during the 6-min walking test were determined. Two daily 8-min sessions of slow–moderate in-home walking at increasing metronome-paced speed were prescribed. After excluding patients with unmeasurable ABI or incompletion of the program, 239 patients were studied. Safe and satisfactory (88%) execution of the prescribed training sessions was reported. During the visits, bilateral ABI improved (+ 0.07; *p* < 0.001) as well as the segmental pressures in the more impaired limb, with changes already significant after 5 weeks of slow walking. Both systolic and diastolic blood pressure decreased overtime (*F* = 46.52; *p* < 0.001; *F* = 5.52; *p* < 0.001, respectively). 6MWD and PFWD improved (41[0‒73]m *p* < 0.001 and 107[42‒190]m *p* < 0.001, respectively) with associated decrease of walking heart rate (*F* = 15.91; *p* < 0.001) and Physiological Cost Index (*F* = 235.93; *p* < 0.001). The variations of most parameters at different visits correlated to the training load calculated. In a regression model, the PFWD variations directly correlated with rate sessions completed, training load and ABI change and inversely with the baseline value (*R*^2^ = 0.27; *p* < 0.001). In the PAD population studied, moderate pain-free exercise improved ABI with associated progressive functional and cardiovascular changes occurring regardless of subjects characteristics.

**Supplementary Information:**

The online version contains supplementary material available at 10.1007/s11739-021-02827-4.

## Introduction

Physical exercise is an essential component of the management program in peripheral artery disease (PAD) [1, 2], a highly prevalent vascular disease associated to low physical function and high risk of cardiovascular events [3, 4]. Recommended exercise programs carried out under supervision three-weekly over 6–8 weeks at an intensity such as to evoke moderate-to-severe pain, are effective at improving walking ability [4–6].

These improvements are attributed to muscle adaptations, walking economy or greater accommodation to pain [4, 7] in absence of reported ankle-brachial index (ABI) or collateral blood flow changes [7, 8]. Various factors related to the hemodynamic picture have been hypothesized to explain this missed outcome [7]. However, vascular adaptations might theoretically occur in PAD patients engaged in exercise programs [7] in the light of the well-described effects of exercise training on vasculature [9–12]. Interestingly, hemodynamic improvements have been observed following pain-free home-based exercise program [13–18] based on the FITT (frequency, intensity, time, type) principles which are differently combined with respect to the recommended programs [4, 19]. This structured walking intervention [13–15, 20, 21], designed to minimize lactate accumulation and favor aerobic adaptations in the ischemic regions [16, 17] was translated into a clinical program. The so-called Test in–Train out (Ti–To) program is based on serial controls at hospital where adherence, patient’s mobility and hemodynamics are assessed.

The hypothesis is that in a population of patients with claudication during the course of this program hemodynamic dose-dependent adaptations are observable with associated functional improvements.

The study aims to describe time course and extent of the effects associated to the progressive training load, in a real-world population of PAD patients consecutively enrolled in a structured rehabilitative program.

## Methods

This is an observational study conducted at Unit of Rehabilitation Medicine at University Hospital of Ferrara. The Ethics Committee CE-AVEC approved the study (277/Oss). The study is reported according to the STROBE statement guidelines.

### Subjects

For the purpose of the study, between January 2015 and December 2019, 459 consecutive patients were screened from Vascular Surgery and enrolled in the rehabilitative program which is available free of charge for PAD patients. The program receives PAD patients at Leriche-Fontaine stage II‒IV, able to walk unassisted or with the habitual device for at least 10 m and without severe cardio-respiratory conditions contraindicating exercise (e.g. unstable angina).

For this study, patients were excluded in case of PAD at stage III–IV; unmeasurable–unreliable ABI (≥ 1.4) [5]; non-completion of the 6-month exercise program for personal or health reasons.

### Intervention

**Fig. 1:**
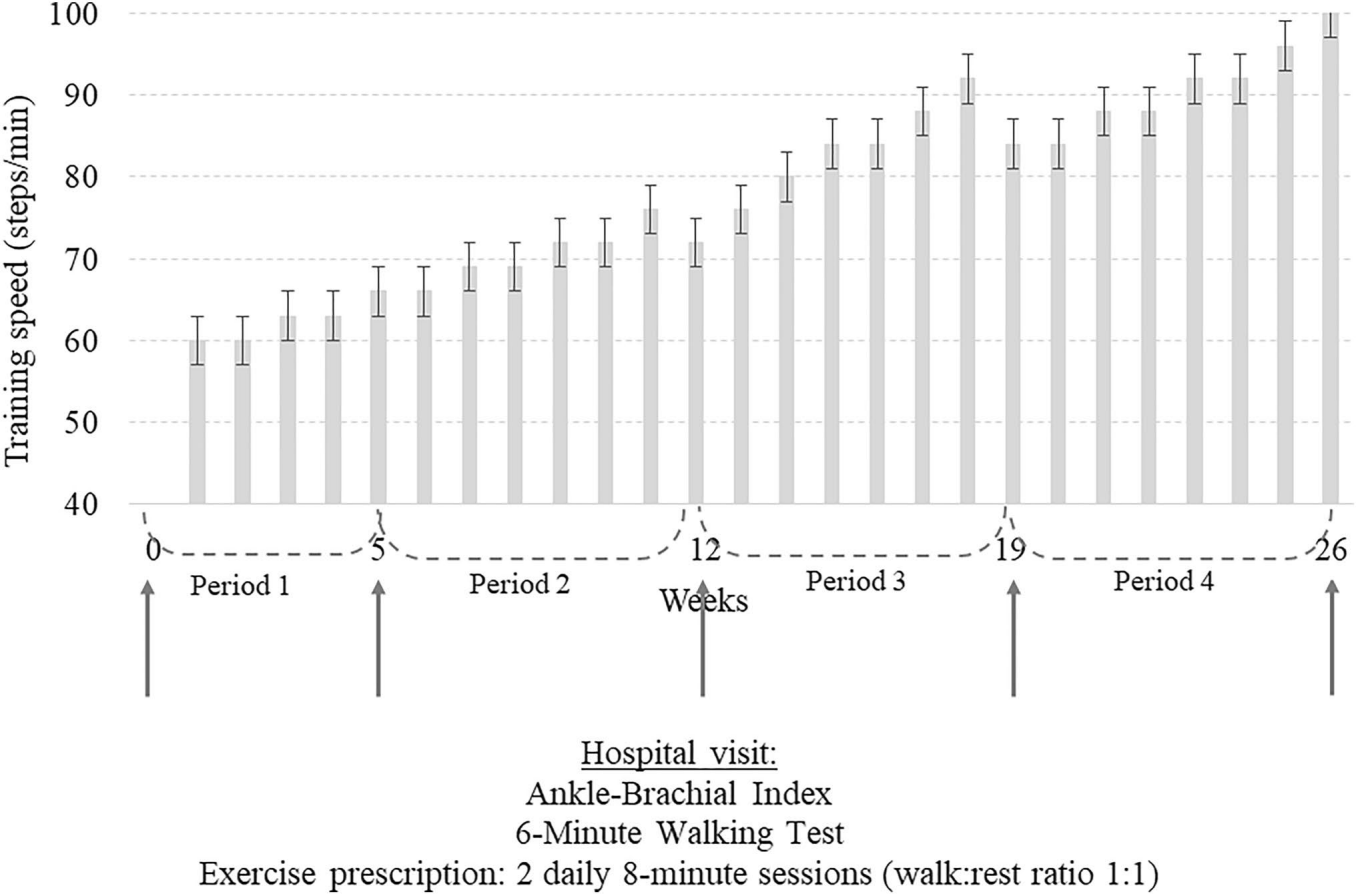
Schematic representation of the training program

All patients were enrolled in the Ti–To program that was prescribed to the patients during five consecutive hospital visits (baseline or week 0, weeks 5 ± 1; 12 ± 1; 19 ± 1; 26 ± 2). The program, fully executed at home, includes two 8-min sessions/day (6 days/week) of intermittent walking (1-min work and 1-min rest while seated) at controlled speed. The prescribed speed, converted into a walking cadence, is paced at home using a metronome (digital or in form of a smartphone application). Exercise is preferably performed inside home (e.g. in a hallway) to reduce possible barriers to exercise (weather, traffic, limited time, fear to fall, etc.) The program, updated at each hospital visit, is reported in Fig. 1.

A training diary to be returned at the subsequent visits is handed to each patient to note the completion of exercise and any possible associated symptoms. The rehabilitation team, composed of a physician and a sports science expert, is available to the patients throughout the entire study period via phone. More details about the exercise program protocol and execution are reported elsewhere [14, 15].

### Study variables

The following variables were collected at baseline and at every hospital visit (T0, T1, T2, T3, T4), by the same skilled operators in a temperature-controlled environment in the morning between 9:00 and 12:00 AM. The same time of measurement was kept for each patient throughout the entire observation.

### Hemodynamics

Ankle-brachial index was measured according to the published standard [5], with the patient laying down in supine position and after 5 min of rest, using Doppler ultrasound transducer (Dopplex SD2, Huntleigh Healthcare Ltd. Diagnostics, Cardiff, United Kingdom) and a standard blood pressure cuff. Blood pressure was measured and recorded at both the posterior tibial arteries (PTA) and dorsalis pedis arteries (DPA) of both limbs. Systolic and diastolic blood pressures at both arms were also assessed.

### Functional capacity

The 6-min walking test (6MWT) was administered during all visits in the same 20-m corridor. Patients were instructed to walk as far as possible for 6 min, with the possibility to rest and restart in case of impossibility to continue walking. The distance at the onset of symptoms referred (pain-free walking distance, PFWD) and the total distance covered (6-min walking distance, 6MWD) were recorded. The habitual speed of each patient was also measured in steps per minutes during the first minute of the test.

The heart rate (HR) was recorded by a pulse oximeter connected via wireless to a smartphone (iHealth, Paris, France) before the test with the patient standing, and during the 6MWT.

At the end, the mean HR and the increasing of HR during the test (ΔHR) were determined. The Physiologic cost index (PCI), or the oxygen expenditure during walking, was also calculated in beats per meter as the ratio between the difference of mean and resting HR and the mean walking speed measured during the 6MWT.

### Training features

The total training load was calculated according to the FITT components [22]. Frequency was the number of weekly sessions reported in the diary, Time was the minutes prescribed for the session (or the actual value reported on the diary in case of incomplete execution). The Intensity, expressed as relative intensity, was the ratio between the prescribed speed (steps/min) and the patient’s habitual walking speed assessed during the baseline 6MWT (e.g.: prescription = 60 steps/min; habitual speed = 100 steps/min; relative intensity = 60/100 = 0.6).

The training load was calculated per week as follows: days/week * min/day * steps/min * relative intensity. For each period, the training load was calculated by summing up the weekly loads.

### Statistical analyses

Data distribution were verified by a Shapiro–Wilk test. Overtime comparison of all variables was performed through a repeated-measures analysis of variance or a Freidman test according to data distribution. The variations between each time point was verified by a paired-samples Wilcoxon test.

Rank correlations between study variables were obtained with a Spearman rho. Univariate regression analyses were conducted to determine the relationship between hemodynamic and performance variables and the total training load for each period. Multiple regression analyses with a forward method of selection were carried out to determine the impact on dependent variables (variations of ABI, PFWD, SPB and segmental pressures) of baseline characteristics of participants and variations of hemodynamics and performance parameters. No missing data were present in the dataset.

A *p* value < 0.05 was considered as significant. Statistical analyses were performed with MedCalc^®^ Statistical Software version 19.6 (MedCalc Software Ltd, Ostend, Belgium).

## Results

Four-hundred and fifty-nine PAD patients were screened and enrolled in the rehabilitation program. For the purpose of this study, 220 patients were excluded for the following reasons: incompressible vessels (*n* = 173), incomplete program execution for health or personal reasons (*n* = 47). A final sample of 239 patients was analyzed.

The anthropometric and clinical characteristics of the population that completed the program are reported in Table 1.

**Table 1:** Characteristic of the population included in the study

	Analyzed (*n* = 239)
Age (years)	72 ± 8
Males, *n* (%)	185 (77)
Education, *n* (%)	
Elementary school	103 (43)
Inferior middle school	103 (43)
Superior middle school	26 (11)
Degree	7 (3)
Risk factors, *n* (%)	
Smoking	209 (87)
Current smoking	23 (10)
Obesity	88 (37)
Hypertension	211 (88)
Hyperlipidaemia	180 (75)
Diabetes	107 (45)
Chronic kidney disease	53 (22)
Comorbidities, *n* (%)	
Coronary artery disease	91 (38)
Cerebrovascular disease	31 (13)
Osteoarticular disease	62 (26)
Rheumatic diseases	12 (5)
Chronic-obstructive pulmonary disease	25 (10)
Age-adjusted Charlson Comorbidity Index	6 ± 2
Peripheral artery disease	
Disease duration (years)	5 ± 5
Lower limb revascularization	27 (28)
Leriche-Fontaine Stage IIa	118 (49)
Leriche-Fontaine Stage IIb	122 (51)
ABI more affected limb	0.66 ± 0.22
ABI less affected limb	0.86 ± 0.21
Pain-free walking distance (m)	114 ± 70
6-min walking distance (m)	288 ± 97

*ABI* ankle-brachial index

All patients included in the analyses safely executed the exercise program without any adverse events related to the training sessions. Patients reported in the diaries a median execution of the 88% (interquartile range 75‒100%) of the training session prescribed. Training features are reported in Online Resource 1.

### Hemodynamic parameters

Ankle-brachial index of the most affected limb progressively improved (*F* = 19.71; *p* < 0.001) from T0 to T4, with significant differences observed between the various time points (Table 2).

**Table 2:** Overtime values of hemodynamic and functional parameters under study

	Week 0	Week 5	Week 12	Week 19	Week 26
More impaired limb					
ABI	0.66 (0.63–0.69)	0.70* (0.67–0.73)	0.71* (0.68–0.74)	0.72*^,^**^,†^ (0.69–0.76)	0.73*^,^**^,†^ (0.70–0.76)
PTA pressure, mmHg	96 (91–101)	97 (92–102)	95 (90–100)	99 (94–104)	100* (95–105)
DPA pressure, mmHg	85 (79–91)	92* (87–98)	94* (89–99)	95* (90–100)	97* (92–102)
Less impaired limb					
ABI	0.86 (0.83–0.89)	0.89 (0.86–0.92)	0.88 (0.85–0.91)	0.89 (0.86–0.92)	0.91 (0.88–0.94)
PTA pressure, mmHg	128 (123–134)	127 (122–131)	123* (119–128)	123* (119–128)	125 (120–130)
DPA pressure, mmHg	123 (118–128)	124 (118–129)	120 (115–125)	120 (116–125)	122 (117–127)
PFWD, m	114 (105–123)	174* (161–186)	207*^,^** (193–220)	224*^,^**^,†^ (210–238)	238*^,^**^,†,‡^ (223–253)
6MWD, m	288 (275–300)	299 (287–310)	316*^,^** (304–328)	324*^,^** (312–337)	328*^,^** (316–340)
SBP, mmHg	159 (156–162)	150* (148–153)	148 * (146–151)	147*^,^** (144–149)	147*^,^** (144–149)
DBP, mmHg	76 (75–77)	74* (73–75)	74* (73–75)	74* (73–75)	74* (73–75)
∆HR, bpm	10 (10–11)	8* (7–8)	7*^,^** (6–7)	6*^,^**^,†^ (6–6)	5*^,^**^,†,‡^ (5–6)
PCI	0.25 (0.23–0.26)	0.17* (0.16–0.18)	0.14*^,^** (0.13–0.15)	0.12*^,^**^,†^ (0.11–0.13)	0.11*^,^**^,†,‡^ (0.10–0.12)

*ABI* ankle-brachial index; *PTA* posterior tibial artery; *DPA* dorsalis pedis artery; *PFWD* pain-free walking distance; *6MWD* 6-min walking distance; *SBP* systolic blood pressure; *DBP* diastolic blood pressure; *HR* heart rate; *PCI* physiological cost index

^*^Different from W0; ** different from W5; ^†^ different from W12; ^‡^ different from W19. Data are expressed as mean (95% confidence interval)

A similar trend was observed also for the contralateral limb, that significantly improved (*F* = 8.73; *p* < 0.001) from T0 to all the other time points.

**Fig. 2:**
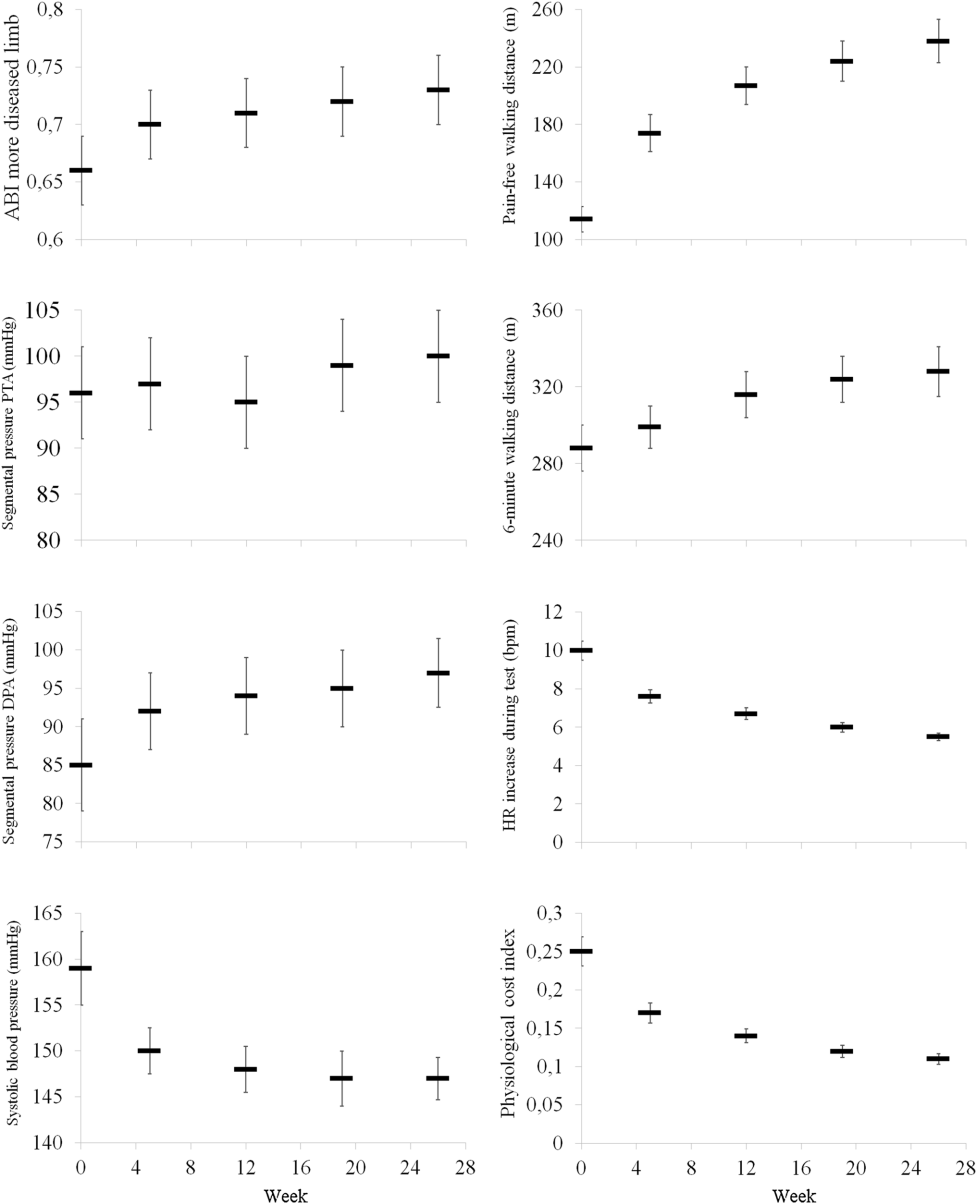
Time course adaptations of hemodynamic and functional parameters during the program. Data are expressed as mean and 95% confidence interval. *PTA* posterior tibial artery; *DPA* dorsalis pedis artery

In the more impaired limb, segmental pressure values also improved (DPA, *F* = 3.08; *p* = 0.015; PTA *F* = 2.60; *p* = 0.035) (Table 2, Fig. 2).

At the contralateral limb for both arteries, a stable non-significant trend was observed.

For arm pressure, SBP exhibited a marked overtime decrease (*F* = 46.52; *p* < 0.001) with significant differences between T0 and all subsequent visits and DBP also significantly decreased (*F* = 5.52; *p* < 0.001) (Table 2, Fig. 2).

### Functional parameters

In the whole population, a median variation of 6MWD of 41 (interquartile range 0‒73) meters was observed. From T0 to the end of the program the 6MWD showed a positive trend (*F* = 58.81; *p* < 0.001) with significant differences observed between the first two visits (T0 and T1) and the remaining ones.

Pain-free walking distance showed a variation of 107 (42‒190) meters from baseline to the end. A greater trend was recorded (*F* = 203.56; *p* < 0.001) with any time point which was significantly different from the other ones.

Resting HR values were stable, while mean HR progressively decreased (*F* = 15.91; *p* < 0.001) with values recorded at T0 significantly different from all the other visits. Accordingly, also the ∆HR during the test was progressively reduced (*F* = 173.33; *p* < 0.001) with significant differences within each time point.

Physiological cost index confirmed the decreasing trend (*F* = 235.93; *p* < 0.001) with an over 100% reduction from T0 to T4 and significant differences between all the time points. (Table 2, Fig. 2).

### Analyses for subgroup of patients

Superimposable hemodynamic and functional results were observed when classifying the PAD population for disease severity (baseline ABI < 0.5 in the more impaired limb), presence of diabetes or sex. All groups showed similar favorable hemodynamics and functional adaptations since T1 until the end of the program (Online Resource 2).

### Relationship between hemodynamic, functional and FITT parameters

At baseline, hemodynamic and performance parameters were significantly correlated. In particular, the 6MWD was related, considering the more impaired and less impaired limb, respectively, to ABI (*r* = 0.23; *p* < 0.001; *r* = 0.23; *p* < 0.001) and to segmental pressure at PTA (*r* = 0.25; *p* < 0.001; *r* = 0.24; *p* < 0.001) and DPA (*r* = 0.18; *p* = 0.006; *r* = 0.27; *p* < 0.001).

**Fig. 3:**
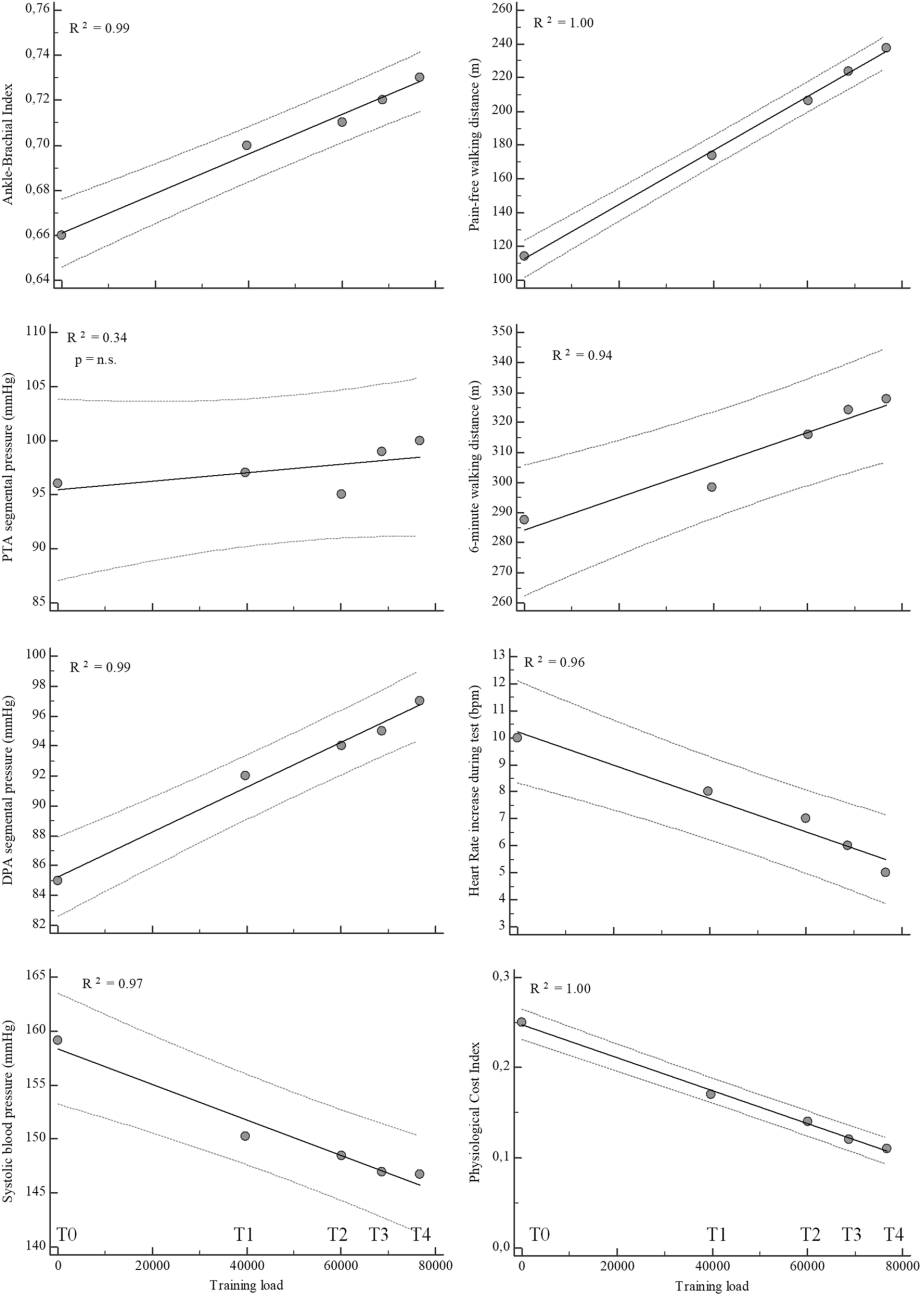
Relation between training load for each period and hemodynamic and functional parameters. Regression line, bold; 95% confidence interval lines, dashed. *PTA* posterior tibial artery; *DPA *dorsalis pedis artery

During the exercise, the training load was significantly related to the values overtime of the hemodynamic and functional parameters. In particular, significant direct correlations were observed for ABI and segmental pressure at DPA of the more impaired limb, PFWD and 6MWD, with congruent negative correlations for SPB, ΔHR and PCI (Fig. 3).

In a significant regression model, the variations of PFWD (*R*^2^ = 0.27; *p* < 0.001) were related with the baseline value (partial *r* = ‒0.35), adherence (partial *r* = 0.15), total training load (partial *r* = 0.24) and variations of ABI of the more impaired limb (partial *r* = 0.18).

## Discussion

The study shows that pain-free exercise induces ABI changes in PAD patients as measured during a progressive program. These adaptations, with associated functional changes, are related with the training load regardless of disease severity, walking ability or sex of patients.

For the first time to our knowledge, the study describes the hemodynamic response to a structured progressive training in a real-world population of PAD patients with claudication enrolled in a home-based program.

Ankle-brachial index changes at discharge of rehabilitation, not reported following the recommended SET [1, 6], as uncommon report were previously highlighted by our research group following TiTo pain-free aerobic program [14–18] and supported by variations of near-infrared spectroscopy (NIRS)-based markers [25, 26]. The study confirms these observations, with an exercise-associated leveled improvement among patients. Its magnitude, corresponding to the decrease of ABI observed in a PAD population in a 4.6 years period [23], is also associated to a lower risk of revascularization at 3 years from discharge of the program [24]. Interestingly, more favorable variations, up to 20% of the basal value, were observed in the patients at more severe hemodynamic status and impaired mobility, where exercise may have contributed to reverse vascular deconditioning related to the disuse [25].

However, hemodynamic improvement after exercise training, even unusual or not reported [6] in PAD patients enrolled in exercise programs, should be expected. It is known that exercise and muscle contractions evoke structural vascular remodeling with different shear stress dependent and independent mechanisms, with nitric oxide-mediated or hypoxia induced effects and with different vascular targets [26, 27]. A possible result is an arteriogenetic response with enlargement of existing arterial vessels and increased blood flow capacity [7, 28].

Furthermore, the kinetics of the onset of adaptations and the relationship with the intensity of exercise and with walking performance represent further important issues for discussion.

For the first point, we highlight that already at the first follow-up visit, after 5 weeks, corresponding to the very slow-speed phase of the program (60–66 steps/min or 1.5–2.0 km h^−1^), the ABI value is significantly higher than baseline.

A training slower than the habitual walking speed, not evoking a critical ischemia in the muscle might therefore represent the key issue for favoring hemodynamic adaptations in the less perfused regions [13, 14]. Notably, this approach differs completely from the training recommended based on faster walking speed, longer bouts of exercise, rest according to patients’ sensation after tolerating ischemic pain [4, 6, 19]. A dose–response effect of exercise on vessels has also been reported [29], with a favorable effect on the endothelial function deriving from low- to moderate-intensity exercise in murine models and in humans [29–31] and recently specifically in PAD patients after submaximal training [9]. Lower inflammation and oxidative stress induced by moderate intensity may contribute, unlike high-intensity exercise [29, 31], also followed by a decrease in vascular function immediately post-exercise [32], by an increase of all blood inflammatory markers in PAD patients and of reactive oxygen species production [9, 33].

Positive changes in vascular tone and endothelial function may also account for the blood pressure variations [34]. In our study, a highly significant decrease in systolic pressure occurs, which drops by nearly 15 mmHg over the course of the program. This fact is relevant in general, and more in PAD patients where an exaggerated BP response to exercise has been observed with an increased cardiovascular risk linked with endothelial dysfunction and arterial stiffness [35–37]. Again, a significant drop in systolic pressure is observed after the first 5 weeks, in the early, slow-speed phase. Such response of the systolic pressure to exercise training was previously reported [38] in particular in hypertensive subjects (5–12 mmHg). This benefit in some cases, especially but non-exclusively in animal models, was associated to low–moderate-intensity exercise [39]. The reported systolic pressure decrease does not diminish the significance of the increase of ABI, considering that in the worst limb the segmental pressure did not decrease at the posterior tibial artery and significantly increased—between 8 and 10% from baseline—at the dorsal artery of the foot.

The training stimulus offered by the program seems effective on vascular adaptations. The intensity of exercise namely the walking speed, is apparently low but matches the limits of energy sustainability in the ischemic regions considering the early deoxygenation occurring in the muscles of PAD patients [40]. The fixed brief walking time and equal standardized passive recovery, avoid a progressive energetic default and a muscular damage related to repeated bouts of ischemia–reperfusion [41]. This aspect is even more important in patients with diabetes where the late perception of ischemic symptoms has been reported [42]. Furthermore, pain-free exercise together with other factors favored the adherence [15, 24, 43–45], with patients at different hemodynamic picture and physical capabilities performing a similar training load from 135 to 155 km/6 months.

The next issue is whether the ABI changes have an impact on the walking capabilities. Despite a previous reported lack of correlation [4], in this study, we observed a relationship between ABI and 6MWD at baseline. As a physiological consequence, a significant relationship between the increase in ABI of the worst limb and changes of the most “aerobic-related” walking distance PFWD were observed after pain-free exercise. In the significant regression model the ABI changes represent a relative percentage of the variations of PFWD, with adherence, training load and baseline PFWD accounting for around 30% of its changes. After all, several exercise-related factors may contribute to the aerobic performance in terms of oxygen transport, delivery and exchange [26, 46]. The angiogenetic response with formation of new capillaries and changes of microvascular function within the muscle may play a role [28]. Notably, selective increase in angiogenetic factors or microvascular density [47, 48], were associated to low-to-moderate-intensity aerobic exercise unlike intense exercise, which represent a weaker stimulus for angiogenesis [48, 49]. A lower sympathetic activation of the arteriolar tone, typical of the low–moderate-intensity exercise, [50, 51] might reduce endothelial dependent capillary reactivity [48]. Finally, adaptations in mitochondrial function may account for the functional improvement. In PAD, such adaptations were reported after an oxygen-guided exercise training assisted by NIRS [52] as well an increase of biomarkers referable to oxygen extraction was documented by NIRS following the program here discussed [17].

As further highlight, considering these potential benefits on the whole aerobic machinery, an increase in 6MWD in a meaningful range [53] and similar to other studies [4] has been observed together with a highly significant change, largely exceeding the large minimal clinically important difference [54] for PFWD. The changes in terms of walking HR with a decreased cardiovascular load and of PCI also support an increased aerobic energy availability with lower cardiac strain.

We underline that the results are limited to the study population. In particular, 36% of the enrolled patients were excluded from the analyses for incompressible arteries and/or unreliable ABI (on the basis of a 66% of subjects that are affected at least by diabetes or chronic kidney disease). This aspect may be related to the real-world design of the study, however, the final sample still included a 45% of patients with diabetes with ABI measurable and progressively improving throughout the program (Online Resource 2).

The study is also limited at patients at Fontaine’s stage II to report the results of a homogenous population in which exercise therapy is recommended by the guidelines. However, in our clinical practice, we observed similar responses to the program also in patients at Fontaine’s stages III and IV able to walk.

The study presents several limitations. First, the retrospective analyses despite a prospectively collected dataset and the absence of a control group. In addition, the outpatient condition of blood pressure data collection may have influenced the results. ABI was not simultaneously arm-limb measured, however, the same expert operators performed all the measurements for the patients included in the study. The proportion of patients lost to vascular stiffness is significant, but the goal was to have determinable values on which to base the study. We, therefore, cannot claim that the same adaptive responses occurred in that subpopulation of patients. Finally, the training load was calculated on reported diaries and not on objectively measured data.

In conclusion, the study in a real-world population supports the concept that hemodynamic response can occur and be quantified in patients with intermittent claudication engaged in exercise training. The proper combination of intensity, duration and frequency of the training bouts to maintain a pain-free exercise is the possible key to induce profitable hemodynamic adaptations in the ischemic districts. However, this intriguing concept referred to a challenging model of performance represented by the PAD patient needs to be confirmed in a larger prospective trial.

## Supplementary Information

Below is the link to the electronic supplementary material.

Supplementary file1 (DOCX 14 KB)

Supplementary file2 (DOCX 123 KB)

## Data Availability

The datasets analyzed during the current study is publicly available at: http://dx.doi.org/10.17632/ssncrycg7m.1.
