# Supplementary material for: Structured pain-free exercise progressively improves ankle-brachial index and walking ability in patients with claudication and compressible arteries: an observational study
Source: Intern Emerg Med. 2021 Sep 9;17(2):439–49. doi: 10.1007/s11739-021-02827-4 (PMC8964614; doi:10.1007/s11739-021-02827-4)
Supplement: Supplementary file 1 — Supplementary file1 (DOCX 14 KB) [file 11739_2021_2827_MOESM1_ESM.docx]

**Online resource 1**

Training features in the four periods under study.

|  | Period 1  (week 1‒5) | Period 2  (week 6‒12) | Period 3  (week 13‒19) | Period 4  (week 20‒26) |
| --- | --- | --- | --- | --- |
| Walking speed (steps/min) | 63±1 | 70±4 | 78±4 | 83±5 |
| % Habitual walking speed | 63% | 70% | 78% | 82% |
| Training load (a.u.) | 25,185 | 36,865 | 52,816; | 63,340 |

Abbreviations: a.u., arbitrary units
