# Supplementary material for: Structured pain-free exercise progressively improves ankle-brachial index and walking ability in patients with claudication and compressible arteries: an observational study
Source: Intern Emerg Med. 2021 Sep 9;17(2):439–49. doi: 10.1007/s11739-021-02827-4 (PMC8964614; doi:10.1007/s11739-021-02827-4)

**Online resource 2**

Time course adaptations of ankle-brachial index (ABI) of the more impaired limb and pain-free walking distance (PFWD) for patients at different: PAD severity (Red: ABI<0.50), diabetes presence (Green: diabetes patients) or sex (Pink: females).

Legend: * p<0.05 respect to baseline


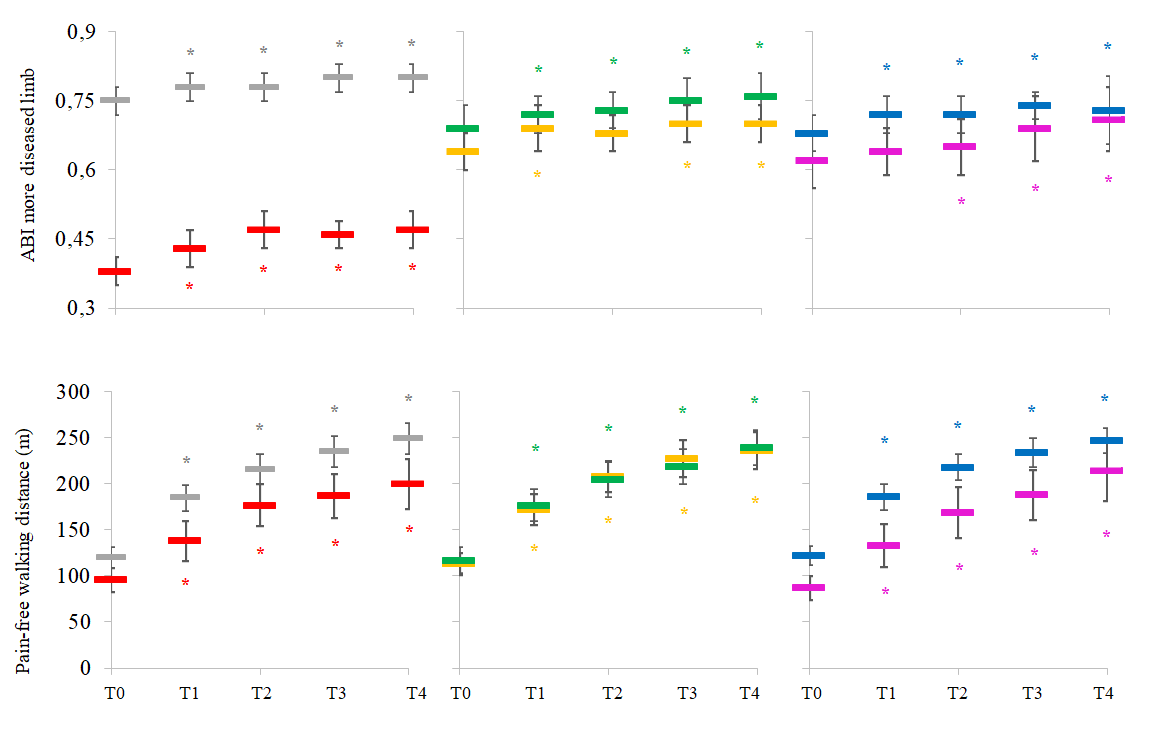

Supplement: Supplementary file 2 — Supplementary file2 (DOCX 123 KB) [file 11739_2021_2827_MOESM2_ESM.docx]
